# The Short-Day Cycle Induces Intestinal Epithelial Purine Metabolism Imbalance and Hepatic Disfunctions in Antibiotic-Mediated Gut Microbiota Perturbation Mice

**DOI:** 10.3390/ijms23116008

**Published:** 2022-05-26

**Authors:** Yongkang Zhen, Yifei Chen, Ling Ge, Wenjun Wei, Yusu Wang, Liangyu Hu, Juan J. Loor, Mengzhi Wang, Junliang Yin

**Affiliations:** 1 College of Animal Science and Technology, Yangzhou University, Yangzhou 225009, China; yongkangzhenyzu@163.com (Y.Z.); cyfjaml@gmail.com (Y.C.); gl1024winnie@163.com (L.G.); weiwennjunn@163.com (W.W.); wyssusu67@163.com (Y.W.); liangyu.hu@wur.com (L.H.); 2 State Key Laboratory of Sheep Genetic Improvement and Healthy Production, Xinjiang Academy of Agricultural Reclamation Sciences, Shihezi 832000, China; 3 Human and Animal Physiology, Wageningen University & Research, 6708 WD Wageningen, The Netherlands; 4 Mammalian Nutrition Physiology Genomics, Department of Animal Sciences, Division of Nutritional Sciences, University of Illinois, Urbana, IL 61801, USA; jloor@illinois.edu

**Keywords:** light–dark cycles, antibiotics, gut microbes, metabolomics, purine metabolism, transcriptome sequencing

## Abstract

Intestinal microbiota dysbiosis is related to many metabolic diseases in human health. Meanwhile, as an irregular environmental light–dark (LD) cycle, short day (SD) may induce host circadian rhythm disturbances and worsen the risks of gut dysbiosis. Herein, we investigated how LD cycles regulate intestinal metabolism upon the destruction of gut microbes with antibiotic treatments. The growth indices, serum parameters, concentrations of short-chain fatty acids (SCFAs), and relative abundance of intestinal microbes were measured after euthanasia; intestinal contents, epithelial metabolomics, and hepatic transcriptome sequencing were also assessed. Compared with a normal LD cycle (NLD), SD increased the body weight, spleen weight, and serum concentration of aspartate aminotransferase, while it decreased high-density lipoprotein. Meanwhile, SD increased the relative abundance of the Bacteroidetes phylum while it decreased the Firmicutes phylum in the gut of ABX mice, thus leading to a disorder of SCFA metabolism. Metabolomics data revealed that SD exposure altered gut microbial metabolism in ABX mice, which also displayed more serious alterations in the gut epithelium. In addition, most differentially expressed metabolites were decreased, especially the purine metabolism pathway in epithelial tissue. This response was mainly due to the down-regulation of adenine, inosine, deoxyguanosine, adenylsuccinic acid, hypoxanthine, GDP, IMP, GMP, and AMP. Finally, the transcriptome data also indicated that SD has some negative effects on hepatic metabolism and endocrine, digestive, and disease processes. Overall, SD induced an epithelial and hepatic purine metabolism pathway imbalance in ABX mice, as well as the gut microbes and their metabolites, all of which could contribute to host metabolism and digestion, endocrine system disorders, and may even cause diseases in the host.

## 1. Introduction

Mammalian biological rhythms regulate physiological activities, habits, nutrient digestion, and metabolism in a 24 h cycle. This system relies on the generation and regulation of the central clock located in the suprachiasmatic nucleus (SCN) of the hypothalamus and the peripheral clock in peripheral organs [1,2]. A stable circadian rhythm is achieved by oscillation in the transcription of genes and their proteins in a process called transcriptional–translational feedback loop (TTFL) [3]. Mechanistically, the transcriptional activators Circadian Locomotor Output Cycles Kaput (CLOCK) and Brain and Muscle ARNT-Like 1 (BMAL1) form a heterodimeric complex in the PER-ARNT-SIM (PAS) domain and then bind to the E-box elements in Cryptochrome (CRY) and Period Circadian Clock (PER) promoters to activate downstream transcription. Period Circadian Clock and CRY proteins enter the nucleus to form PER-CRY oligomers, which inhibit heterodimer activity and transcription [4,5]. The light-dark cycles are the main environmental factor regulating the expression of circadian rhythms, with light acting on intrinsically photosensitive retinal ganglion cells (ipRGCs) to send signals into the SCN to guide periodical oscillations of the central clock and the output of rhythmic behavior consistent with light–dark cycles [6,7].

The circadian rhythms and light–dark cycles regulate a variety of gastrointestinal functions including cell proliferation, immune homeostasis, intestinal permeability, and microbial balance and metabolism, all of which are key to optimal health [8,9,10,11,12]. For example, the circadian rhythm of gut microbes was disrupted and led to metabolic disturbances in *Per1/2*^−/−^ mice [11,12]. In another study, cell proliferation was aberrant and pro-inflammatory cytokine abundance in the gastrointestinal tract was increased in *Bmal1/Clock/Cry* mutant mice [9,10,13]. Mutations in the *CLOCK* gene were also found in colorectal cancer [14]. In humans, circadian rhythm disorders can increase susceptibility to digestive diseases, intestinal inflammation and duodenal ulcers, obesity, and diabetes, in large part because the abnormalities in the sleep and wake cycles increase food intake, especially at night [15,16].

The diversified microbial ecology in the colon and cecum and the intestinal epithelium co-evolved to form a strong immune system and barrier function through the host to promote mutual benefits between host and microbes [17,18]. Gut microbes may contribute to immunity, nerve signal transduction, intestinal endocrine and metabolic health of the human host and, when aberrant, to the pathogenesis of various common metabolic disorders such as obesity, type-two diabetes, non-alcoholic liver disease [19], and mucosal inflammation such as Crohn’s disease [20]. Gut microbes serve as potential biomarkers for many diseases, e.g., down-regulation of *Akkermansia muciniphila*, *Bacteroides thetaiotaomicron*, and *Clostridium histolyticum* is related to obesity. Type-two diabetes was associated with up-regulation of *Bacteroides vulgatus*, *Clostridium clostridioforme*, and *Prevotella copri.* In addition, *Coprococcus comes* and *Faecalibacterium prausnitzii* had a lower abundance during liver disease [19]. In cardiovascular diseases, enrichment of *Streptococcus*, *Escherichia*, and *Collinsella* along with decreased numbers of *Bacteroides* and *Prevotella* were associated with atherosclerosis [21]. Circadian rhythms can also generate innate immunity through microbes, e.g., environmental light–dark cycles entrain circadian feeding behaviors that produce rhythms in producing antimicrobial proteins by gut microbes, and the rhythmic expressions of antimicrobial proteins were driven by daily rhythms in epithelial attachment by segmented filamentous bacteria. As such, these events can further promote corresponding changes in intestinal innate immunity to resist exposure to exogenous microorganisms [22].

Gut microbes are the main force for the digestion of complex carbohydrates in the hindgut. This leads to the production of short-chain fatty acids (SCFAs), vitamins, hormones, amino acid derivatives, and antioxidants, all of which can be directly absorbed by the intestinal epithelium into the host circulatory system and maintain a proper intestinal barrier function. Among the SCFAs, acetate, butyrate, and propionate produced by gut microbes are the most abundant and well-studied ones. They may bind to G protein-coupled receptors (such as GPR41/43) to affect host metabolism [23]. Short-chain fatty acids in the blood may reduce oxidative stress and help maintain immune function [21]. They may also improve glucose metabolism and insulin secretion by inducing the secretion of gut hormones such as glucagon-like peptide 1 (GLP-1) and peptide YY (PYY) [19]. Microbial valerate and butyrate have been associated with enhancing the anti-tumor activity of cytotoxic T lymphocytes (CTL) and chimeric antigen receptor (CAR) T cells, thus improving cancer immunotherapy [24].

In this study, we hypothesized that SD could potentially aggravate gut microbial metabolism disorders and may have some negative impacts on liver functions in gut dysbiosis mice. To address the hypothesis, the negative control (NC) and antibiotic-treated (ABX) mice were managed under normal LD (NLD) and SD cycles, to study the impacts of intestinal contents and epithelium metabolism using metabolomics and the liver functions using transcriptome sequencing. An important goal was to construct an intestinal microbe disorder murine model through the use of antibiotics.

## 2. Results

### 2.1. Validation of the Model of Antibiotic-Induced Gut Dysbiosis in Mice

**Figure 1:**
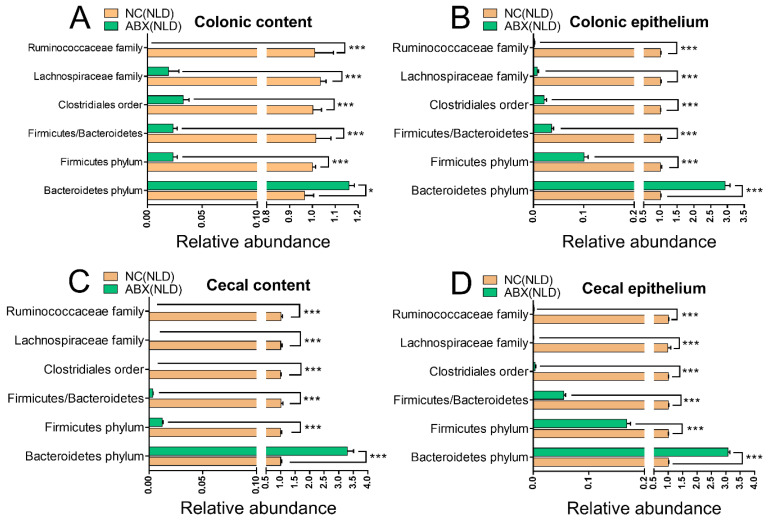
The relative abundance of gut microbiota in colonic and cecal contents and epithelium of NC and ABX mice under NLD. NC (NLD), the negative control mice managed under normal light–dark cycle; ABX (NLD), the antibiotic-treated mice managed under normal light–dark cycle. Representative charts of the relative abundance of Bacteroidetes phylum, Firmicutes phylum, Clostridiales order, Lachnospiraceae family, Ruminococcaceae family, and the ratio of Firmicute to Bacteriodetes in colonic content (**A**), colonic epithelium (**B**), cecal content (**C**), and cecal epithelium (**D**) were detected using RT-PCR, where the 16S rRNA gene amplified by the total bacterial primer set was used as an internal reference, and the abundance of gut microbiota in ABX (NLD) was relative to NC (NLD). Values were means, with standard error of the mean. * *p* < 0.05, *** *p* < 0.001.

The relative abundance of gut microbiota in colonic and cecal contents and epithelium of NC and ABX mice under NLD were measured to validate the efficiency of antibiotic-mediated gut microbiota perturbation. Data indicated that ABX significantly increased the relative abundance of the Bacteroidetes phylum while it decreased the relative abundances of the Firmicutes phylum, Clostridiales order, Lachnospiraceae family, and Ruminococcaceae family in colonic content (Figure 1A), colonic epithelium (Figure 1B), cecal content (Figure 1C), and cecal epithelium (Figure 1D) compared with NC (*p* < 0.05 or *p* < 0.001). Meanwhile, the ratio of Firmicutes to Bacteroidetes was also significantly decreased in ABX compared with NC (*p* < 0.001). In summary, we had a significant effect in establishing the model of gut dysbiosis mice.

### 2.2. Body Mass, Body Temperature, Organ Indexes, and Serum Biochemical Indicators in ABX Mice under NLD and SD

**Figure 2:**
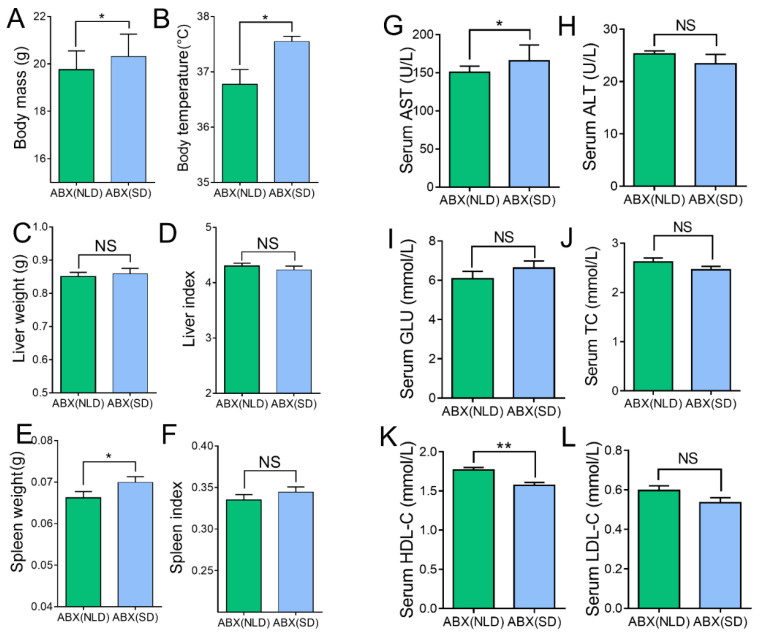
Body mass, body temperature, organ indexes, and serum biochemical indicators in ABX mice under NLD and SD. ABX (NLD), the antibiotic-treated mice managed under normal light–dark cycle; ABX (SD), the antibiotic-treated mice managed under short-day cycle. Representative charts of body weight (**A**), body temperature (**B**), liver weight (**C**), liver index (**D**), spleen weight (**E**), spleen index (**F**), and concentrations of serum AST (**G**), ALT (**H**), GLU (**I**), TC (**J**), HDL-C (**K**), and LDL-C (**L**) were determined in ABX mice under NLD and SD. Values were means, with standard error of the mean. * *p* < 0.05, ** *p* < 0.01, ^NS^ *p* > 0.05.

We next investigated the alterations in body mass, body temperature, organ indexes, and serum biochemical indicators in ABX mice under NLD and SD. Data indicated that SD increased the body mass (Figure 2A), body temperature (Figure 2B), and spleen weight compared with NLD (Figure 2E) (*p* < 0.05); however, no significant differences were observed in indicators such as liver weight (Figure 2C), liver index (Figure 2D), and spleen index (Figure 2F) (*p* > 0.05). In terms of serum indicators, the concentration of serum aspartate aminotransferase (AST) increased (Figure 2G), while the concentration of serum high-density lipoprotein (HDL-C) decreased (Figure 2K) in SD compared with NLD (*p* < 0.05); however, SD had no impacts on the concentrations of serum alanine aminotransferase (ALT) (Figure 2H), glucose (GLU) (Figure 2I), total cholesterol (TC) (Figure 2J), or low-density lipoprotein (LDL-C) (Figure 2L).

### 2.3. Gut Microbiota Profiles in ABX Mice under NLD and SD

**Figure 3:**
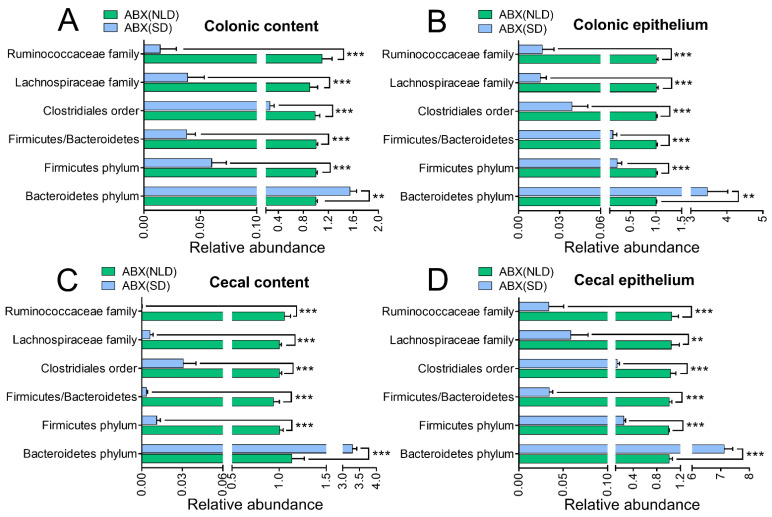
The relative abundance of gut microbiota in colonic and cecal contents and epithelium in ABX mice under NLD and SD. ABX (NLD), the antibiotic-treated mice managed under normal light–dark cycle; ABX (SD), the antibiotic-treated mice managed under short-day cycle. Representative charts of the relative abundance of Bacteroidetes phylum, Firmicutes phylum, Clostridiales order, Lachnospiraceae family, Ruminococcaceae family, and the ratio of Firmicute to Bacteriodetes in colonic content (**A**), colonic epithelium (**B**), cecal content (**C**), and cecal epithelium (**D**) were detected using RT-PCR, where the 16S rRNA gene amplified by the total bacterial primer set was used as an internal reference, and the abundance of gut microbiota in ABX (SD) was relative to ABX (NLD). Values were means, with standard error of the mean. ** *p* < 0.01, *** *p* < 0.001.

Data indicated that SD had a significant impact on the relative abundance of gut microbiota in colonic and cecum contents and epithelium compared with NLD in ABX mice (Figure 3). Compared with NLD, SD significantly increased the relative abundance of the Bacteroidetes phylum, while it decreased the relative abundances of Firmicutes, Clostridiales order, Lachnospiraceae family, and Ruminococcaceae family in colonic content (Figure 3A), colonic epithelium (Figure 3B), cecal content (Figure 3C), and cecal epithelium (Figure 3D) (*p* < 0.01 or *p* < 0.001). Moreover, SD also decreased the ratio of Firmicutes to Bacteroidetes compared with NLD (*p* < 0.001).

### 2.4. The Proportions of SCFAs in Colonic and Cecal Contents of ABX Mice under NLD and SD

**Figure 4:**
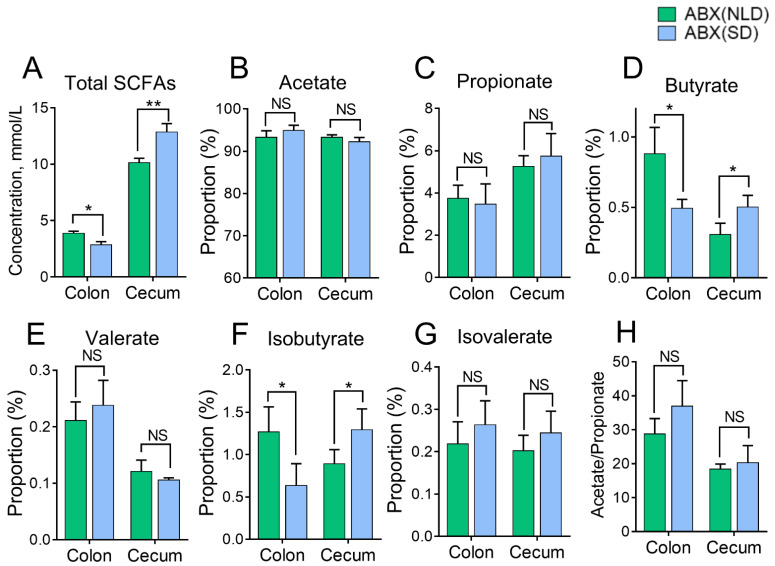
The proportions of SCFAs in colonic and cecal contents of ABX mice under NLD and SD. ABX (NLD), the antibiotic-treated mice managed under normal light–dark cycle; ABX (SD), the antibiotic-treated mice managed under short-day cycle. Representative charts of the concentration of total SCFAs (**A**), and the proportion of acetate (**B**), propionate (**C**), butyrate (**D**), valerate (**E**), isobutyrate (**F**), isovalerate (**G**), and the ratio of acetate to propionate (**H**) were determined in ABX mice under NLD and SD. Values were means, with standard error of the mean. * *p* < 0.05, ** *p* < 0.01, ^NS^ *p* > 0.05.

Compared with NLD, SD significantly decreased the concentrations of total SCFAs (Figure 4A) and the proportions of butyrate (Figure 4D) and isobutyrate (Figure 4F) (*p* < 0.05), but had no impacts on the proportions of acetate (Figure 4B), propionate (Figure 4C), valerate (Figure 4E), isovalerate (Figure 4G), or the ratio of acetate to propionate (Figure 4H) (*p* > 0.05) in colonic contents. In terms of the proportions of SCFAs in cecal contents and the concentration of total SCFAs (Figure 4A), the proportions of butyrate (Figure 4D) and isobutyrate were significantly increased in ABX mice under SD compared with NLD (*p* < 0.05), but had no impacts on the proportions of acetate (Figure 4B), propionate (Figure 4C), valerate (Figure 4E), isovalerate (Figure 4G), or the ratio of acetate to propionate (Figure 4H) (*p* > 0.05).

### 2.5. Overview of Metabolomics Profiles and the Volcano Plots of Differentially Altered Metabolites of Intestinal Contents and Epithelium in ABX Mice under NLD and SD

**Figure 5:**
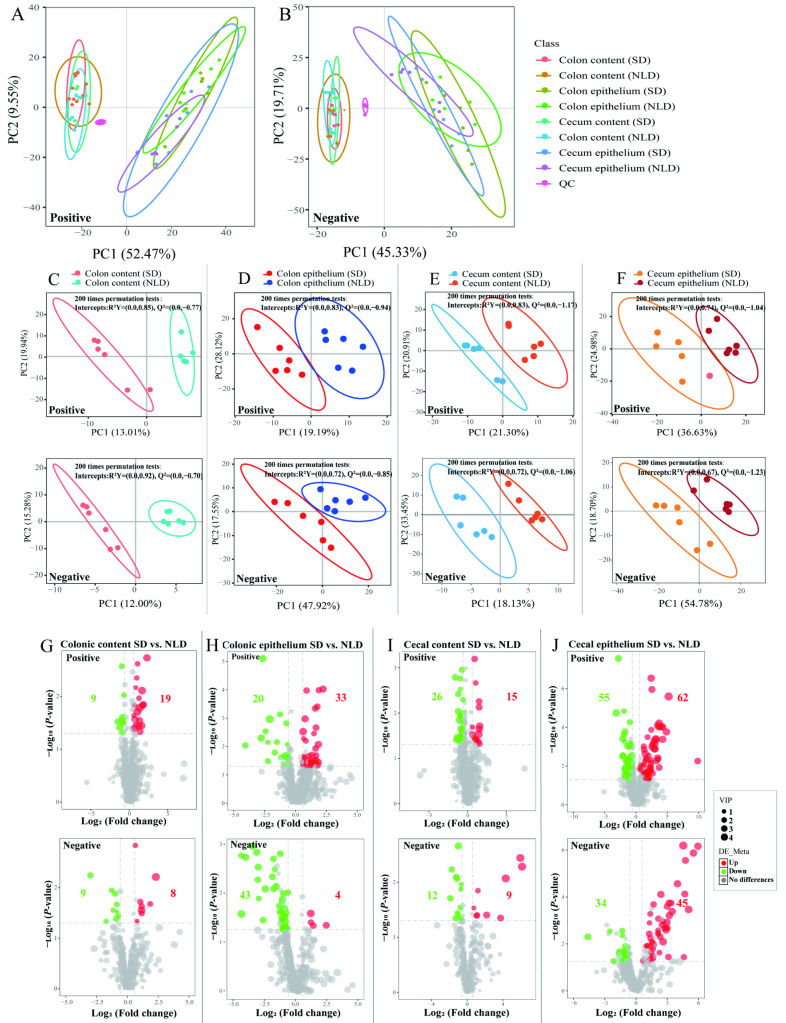
Overview of metabolomics profiles and the volcano plots of significantly different metabolites of colonic and cecal contents and epithelium in ABX mice under NLD and SD. NLD, the antibiotic-treated mice managed under normal light–dark cycle; SD, the antibiotic-treated mice managed under short-day cycle; QC, quality control group. The PCA plot based on LC/MS analysis in ABX mice under NLD and SD among different groups in positive ion mode (**A**) and negative ion mode (**B**) are shown. The PLS-DA plot based on LC/MS analysis and its corresponding validation plots based on 200 times permutation tests in colonic contents (**C**), colonic epithelium (**D**), cecal contents (**E**), and cecal epithelium (**F**) in ABX mice under NLD and SD are shown in both positive and negative ion modes. Volcano plots of differential metabolites in colonic contents (**G**), colonic epithelium (**H**), cecal contents (**I**), and cecal epithelium (**J**) in both positive and negative ion modes are shown in ABX mice under NLD and SD. Red dots represent increased, blue dots represent decreased, and the gray dots represent did not change significantly; numbers represent the number of significant different metabolites.

Distributions of metabolomics data using principal components analysis (PCA) score plots among different groups in positive and negative ion mode were used to discern biologically meaningful patterns. In the positive ion mode, the score plot displayed 52.47% and 9.55% of variables by the PC1 and PC2 axis (Figure 5A), respectively, while in the negative ion mode, the PC1 and PC2 axis displayed variables of 45.33% and 19.71% (Figure 5B), respectively. The comparison demonstrated that the metabolites in colonic and cecal contents were separated from those in epithelium in ABX mice both under SD and NLD (Figure 5A,B).

To further illustrate the contribution of different LD cycles to the classification and differentiation of metabolites in antibiotic-treated mice intestines, the partial least squares discriminant analysis (PLS-DA) score plots were used. Clear separation and discrimination were detected between SD and NLD in both positive and negative ion modes for colonic contents (Figure 5C), colonic epithelium (Figure 5D), cecal contents (Figure 5E), and cecal epithelium (Figure 5F) in ABX mice. Furthermore, the permutation tests were performed to validate the PLS-DA model, and the results after 200 permutations revealed high *R*^2^*Y* (cum) and *Q*^2^ (cum) lower than zero between the SD and NLD of all groups.

Metabolites with VIP > 1 and *p* < 0.05 were identified as differentially altered metabolites. In general, the volcano plots showed that 45 differentially altered metabolites were screened in colonic contents between SD and NLD in ABX mice in both positive and negative ion modes (containing unknown and unidentified metabolites). Of those, compared with NLD, 18 metabolites were decreased and 27 metabolites were increased under SD (Figure 5G); in colonic epithelium, 100 differentially altered metabolites were screened, with 63 decreased and 37 increased under SD compared with NLD (Figure 5H); in cecal content, 62 differentially altered metabolites were screened, with 38 decreased and 24 increased under SD compared with NLD (Figure 5I); and in colonic epithelium, 196 differentially altered metabolites were screened, with 89 decreased and 107 increased under SD compared with NLD (Figure 5J).

### 2.6. Identification and Classification of Differentially Altered Metabolites of Metabolomics Data in ABX Mice under NLD and SD

**Figure 6:**
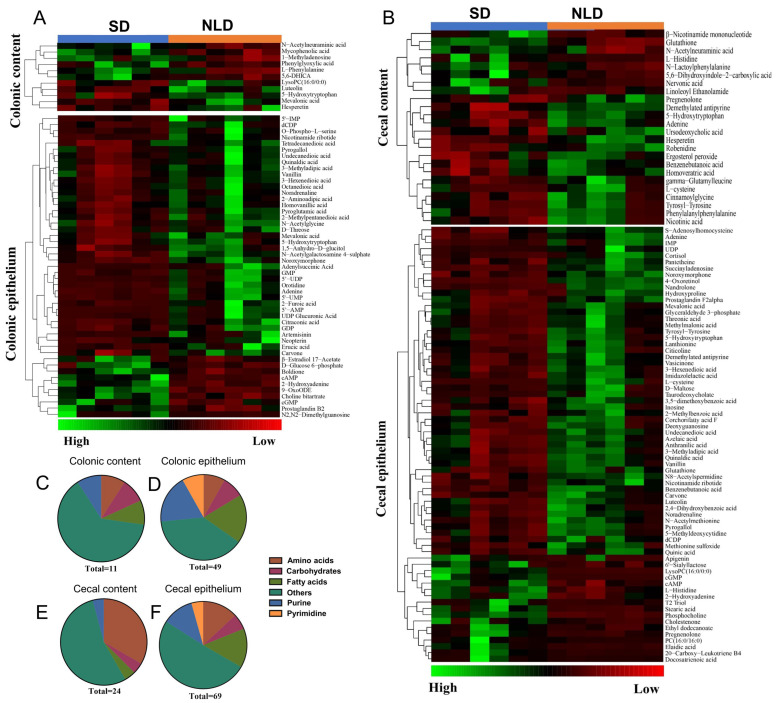
Identification and classification of significantly different metabolites in ABX mice under NLD and SD. NLD, the antibiotic-treated mice managed under normal light–dark cycle; SD, the antibiotic-treated mice managed under short-day cycle. Cluster heatmap of differential metabolites in colonic contents and epithelium (**A**); cecal contents and epithelium (**B**) were shown in ABX mice under NLD and SD. Red color represents low abundance and green color represents high abundance. The proportions of amino acids, carbohydrates, fatty acids, purine, and others of significant different metabolites were classified in colonic contents (**C**), colonic epithelium (**D**), cecal contents (**E**), and cecal epithelium (**F**).

A cluster heatmap was used to identify accurate differentially altered metabolites and obtain pathway classification information. In colonic contents, 11 metabolites were accurately identified between NLD and SD, including L-phenylalanine within amino acid; N-acetylneuraminic acid within carbohydrate; mevalonic acid within fatty acid; and phenylglyoxylic acid within purine (Figure 6A, Table S1); and ~63.64% could not be classified (Figure 6C). In colonic epithelium, 49 metabolites were accurately identified, but nearly 40% could not be classified (Figure 6D) between NLD and SD. This list contained 2-aminoadipic acid, N-acetylglycine, etc., within amino acid; D-threose, D-glucose 6-phosphate, etc., within carbohydrate; erucic acid, citraconic acid, mevalonic acid, etc., within fatty acid; cAMP, cGMP, AMP, GDP, GMP, etc., within purine; and orotidine, UDP, UMP, etc., within pyrimidine (Figure 6A, Table S1). Similarly, 24 metabolites were accurately identified in cecal contents, but nearly 50% could not be classified (Figure 6E) between NLD and SD. The list contained glutathione, L-cysteine, L-histidine, tyrosyl-tyrosine, etc., within amino acid; N-acetylneuraminic acid within carbohydrate; nervonic acid within fatty acid; and adenine within purine (Figure 6B, Table S2). Lastly, glutathione, lanthionine, L-cysteine, L-histidine, etc., within amino acid; D-maltose, threonic acid, etc., within carbohydrate; elaidic acid, mevalonic acid, stearic acid, etc., within fatty acid; adenine, inosine, cAMP, cGMP, etc., within purine; and citicoline, UDP, etc., within pyrimidine were identified in cecal epithelium between NLD and SD (Figure 6B, Table S2), with a total of 69 differentially altered metabolites and ~50% that could not be classified (Figure 6F).

### 2.7. Characterization and Functional Analysis of Key Metabolic Pathways of Metabolomics Data in ABX Mice under NLD and SD

**Figure 7:**
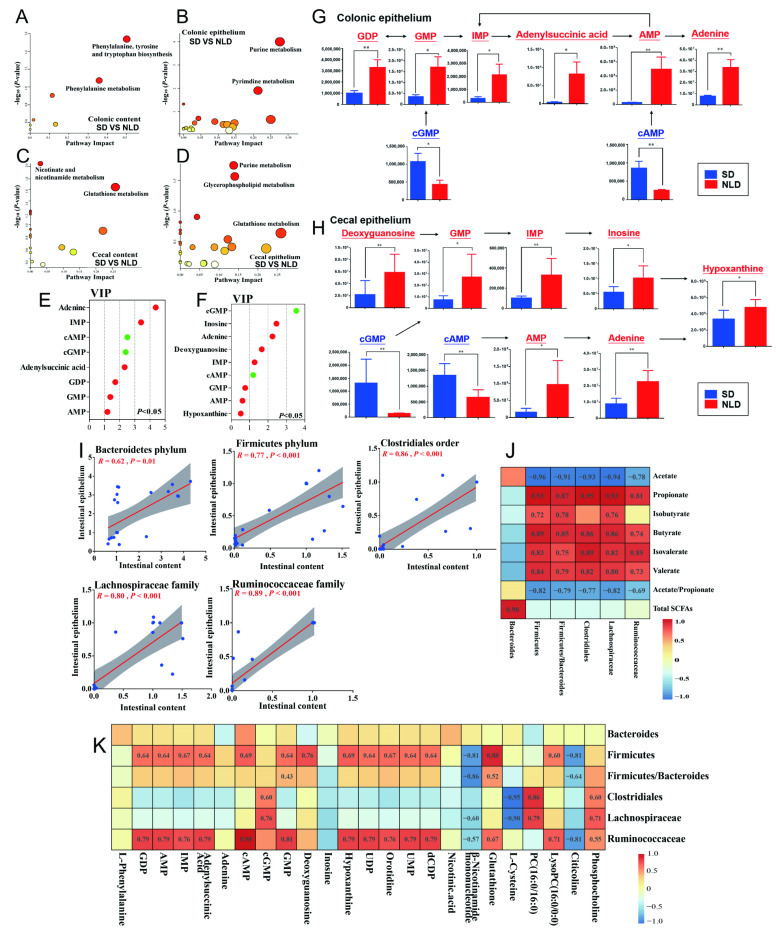
Characterization and functional analysis of key metabolic pathways and correlations between gut microbes, SCFAs proportions, and metabolites. NLD, the antibiotic-treated mice managed under normal light–dark cycle; SD, the antibiotic-treated mice managed under short-day cycle. Pathways were enriched via enrichment and topology analysis of significantly different metabolites in colonic contents (**A**), colonic epithelium (**B**), cecal contents (**C**), and cecal epithelium (**D**) in ABX mice under NLD and SD. Large sizes and red colors represent high pathway impact and major pathway enrichment, while small sizes and other colors represent low pathway impact and minor pathway enrichment. Variable importance in projection (VIP) scores of metabolites in the purine metabolism pathway were also obtained from the PLS-DA models in colonic epithelium (**E**) and cecal epithelium (**F**). Red dots represented decreased and green dots represented increased. Relevant metabolic pathways map based on the proportions of differential metabolites of purine metabolism pathway in colonic epithelium (**G**) and cecal epithelium (**H**), * *p* < 0.05, ** *p* < 0.01. Red represented decreased and blue represented increased. Pearson correlation coefficients between the relative abundance of gut microbes in intestinal contents and epithelium of Bacteroidetes phylum, Firmicutes phylum, Clostridiales order, Lachnospiraceae family, and Ruminococcaceae family (**I**) were analyzed in ABX mice, red line represented the best-fit line and grey region represented the 95% confidence band of the best-fit line. Pearson correlation coefficients between the relative abundance of gut microbes and the proportions of SCFAs (**J**) were analyzed in ABX mice. Pearson correlation coefficients between the relative abundance of gut microbes and the significantly differential metabolites in purine and pyrimidine metabolism pathways (**K**) were also analyzed in ABX mice. The Pearson correlation coefficients (*R*) are shown on the plots; positive correlations are displayed in red and negative correlations are shown in blue, while the (*R*) shown in the grid proved that the correlation reached a significant level, *p* < 0.05.

Data from KEGG analysis showed that, compared with NLD, the increased metabolite L-phenylalanine under SD in colonic contents was enriched in “Phenylalanine, tyrosine and tryptophan biosynthesis” and “Phenylalanine metabolism” pathways (Figure 7A, Table S3). In colonic epithelium, most metabolites were in the “Purine metabolism” pathway, and SD increased cAMP and cGMP while it decreased GDP, AMP, IMP, GMP, adenine, and adenylsuccinic acid compared with NLD. In the “Pyrimidine metabolism” pathway, SD decreased metabolites such as UDP, orotidine, UMP, and dCDP compared with NLD (Figure 7B, Table S3). In cecal contents, SD increased the β-nicotinamide mononucleotide, while it decreased nicotinic acid, and both metabolites were enriched in the “Nicotinate and nicotinamide metabolism” pathway. SD also increased glutathione, while it decreased L-cysteine, and both metabolites were enriched in the “Glutathione metabolism” pathway (Figure 7C, Table S3). Lastly, in cecal epithelium, most metabolites were targeted to “Purine metabolism” (cAMP and cGMP increased while inosine, adenine, deoxyguanosine, and IMP decreased under SD compared with NLD), “Glycerophospholipid metabolism” (PC(16:0/16:0), LysoPC(16:0/0:0)), phosphocholine (increased while citicoline decreased under SD compared with NLD), and “Glutathione metabolism” pathways (glutathione and L-cysteine decreased under SD compared with NLD) (Figure 7D, Table S3).

In colonic epithelium, metabolites enriched in the purine metabolism pathway ranked by VIP value were adenine, IMP, cAMP, cGMP, adenylsuccinic acid, GDP, GMP, and AMP, all of which were decreased except for cAMP and cGMP under SD compared with NLD (Figure 7E). The relevant metabolic pathway map indicated that SD decreased the transformation of GDP to the adenine pathway in colonic epithelium compared with NLD (Figure 7G). Similarly, inosine, adenine, deoxyguanosine, IMP, GMP, AMP, and hypoxanthine were decreased (*p* < 0.05 but VIP < 1 of GMP, AMP, and Hypoxanthine) while cGMP and cAMP were increased in cecal epithelium under SD compared with NLD (Figure 7F). The pathway of adenine and inosine to synthesize hypoxanthine was inhibited under SD in antibiotic-treated mice compared with NLD (Figure 7H). In addition, compared with NLD, SD inhibited the conversion of cGMP to GMP and cAMP to AMP in intestinal epithelium.

### 2.8. Correlations between Gut Microbes, SCFA Proportions, and Metabolites

The relative abundances of the Bacteroidetes phylum, Firmicutes phylum, Clostridiales order, Lachnospiraceae family, and Ruminococcaceae family were used to determine the correlations between gut microbes in intestinal contents and epithelium in ABX mice, together with other metabolites. Figure 7I underscored that the relative abundance of gut microbes in intestinal contents were positively correlated with those in intestinal epithelium, such as the Bacteroidetes phylum (*R* = 0.62, *p* = 0.01), Firmicutes phylum (*R* = 0.77, *p* < 0.001), Clostridiales order (*R* = 0.86, *p* < 0.001), Lachnospiraceae family (*R* = 0.80, *p* < 0.001), and Ruminococcaceae family (*R* = 0.89, *p* < 0.001). In addition, the correlations between the relative abundance of gut microbes and the concentrations of SCFAs were also analyzed (Figure 7J), and data indicated that the concentrations of total SCFAs are positively correlated with the relative abundance of the Bacteroidetes phylum (*R* = 0.90, *p* < 0.05). The proportions of propionate, isobutyrate, butyrate, isovalerate, and valerate were mainly positively correlated with the abundance of the Firmicutes phylum, Clostridiales order, Lachnospiraceae family, Ruminococcaceae family, and the ratio of Firmicutes to Bacteriodetes (*R* > 0.7, *p* < 0.05), while the proportion of acetate and the ratio of acetate to propionate had negative correlations with the gut microbes (*R* < −0.6, *p* < 0.05). Lastly, the metabolites targeted in purine and pyrimidine metabolism pathways were mainly positively correlated with the relative abundance of the Firmicutes phylum and Ruminococcaceae family (Figure 7K), which indicated that down-regulation abundance of Firmicutes may result in the inhibition of purine and pyrimidine metabolism under SD in ABX mice.

### 2.9. Hepatic Transcriptome Sequencing Profiles and Pathways Enrichment in ABX Mice under NLD and SD

**Figure 8:**
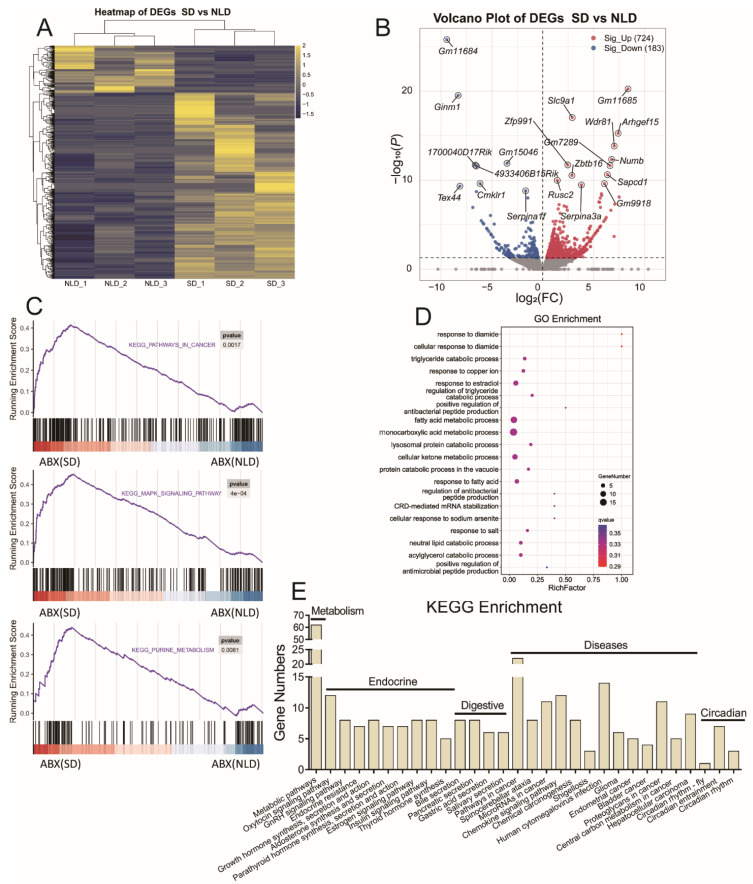
Hepatic transcriptome sequencing profiles and pathway enrichments in ABX mice under NLD and SD. NLD, the antibiotic-treated mice managed under normal light–dark cycle; SD, the antibiotic-treated mice managed under short-day cycle. The differentially expressed genes (DEGs) were screened out by setting *p* < 0.05 and |log 2 Fold Change| > 1 as threshold. (**A**) The heatmap of the differentially expressed genes; (**B**) the volcano plot of the differentially expressed genes, red dots represent up-regulated and blue dots represent down-regulated; (**C**) GSEA plots for the enrichments of pathways in cancer, MAPK signaling pathway, and purine metabolism; (**D**) Gene Ontology (GO) functional analysis of the significantly expressed genes; (**E**) KEGG functional analysis of the significantly expressed genes.

Finally, we investigated the alterations in hepatic functions induced by SD in ABX mice using transcriptome sequencing technology. A total number of 907 differentially expressed genes (DEGs) were selected (Figure 8A and Table S4) using a cut off of *p* < 0.05. A volcano plot was also used to visualize the changes of DEGs in the liver of ABX mice under SD and NLD (Figure 8B). Compared with NLD, 724 DEGs were up-regulated, including *Slc9a1*, *Arhgef15*, *Wdr81*, *Numb*, etc., in SD, while 183 DEGs were down-regulated, including *Ginm1*, *Cmklr1*, *Tex44*, *Serpina1f*, etc., in SD.

To assess the functional consequences, the up-regulated genes and down-regulated genes were analyzed using the KEGG database, Gene Ontology (GO) database, and gene set enrichment analysis (GSEA). We used a GSEA analysis to examine the impacts on SD-mediated hepatic dysfunctions on the transcriptome data. Pathways were significantly enriched in cancer (*p* = 0.0017) and the MAPK signaling pathway (*p* = 0.0004) (Figure 8C). It is interesting to find that SD also impacted the purine metabolism pathway in the liver (*p* = 0.0081) compared with NLD (Figure 8C). Next, enrichment analysis using the GO database showed that most DEGs were enriched in several processes, such as fatty acid metabolic process, monocarboxylic acid metabolic process, and cellular ketone metabolic process (Figure 8D). Finally, the enrichment analysis results of the KEGG database were consistent with those in the GO and GSEA analyses. Short day significantly altered the pathways in metabolism, endocrine system, digestive system, and circadian rhythms compared with NLD and may induce diseases such as cancers (Figure 8E).

## 3. Discussion

Infusion of antibiotics is one common method to induce gut microbe dysbiosis [25,26,27]. Thus, the use of a cocktail containing gentamicin, sulbenicillin, cephalosporin, and amphotericin to deplete the gut microbiota in our study was appropriate to address our objectives. Data indicated that compared with NC mice, the relative abundances of Firmicutes phylum were significantly decreased, while the Bacteroidetes phylum were significantly increased in colonic and cecal contents and epithelium of ABX mice. Several previous studies indicated that the changes in the profiles of intestinal microbes in ABX mice have not been consistent; for example, the relative abundance of the Bacteroidetes phylum was increased, and the Firmicutes phylum decreased with ampicillin, neomycin, metronidazole, vancomycin, and the antifungal amphotericin [25]. In contrast, the relative abundance of the Bacteroidetes phylum decreased, and the Firmicutes phylum increased when animals received lincomycin [26]. The gut microbial colonization and composition were all different in mice receiving ampicillin, metronidazole, or vancomycin treatments [28]. Moreover, the exposure time also significantly affected the relative abundance of each bacterial phyla [29]. We also noticed that SD had a significant reduction in body mass and organ indexes in ABX mice compared with NC, which was consistent with a previous report [26], the reason may be that the infusion of antibiotics led to destroy the colonic barrier function and induce colitis, thus having the poor nutritional status in ABX mice [26]. Another possible reason of dehydration in ABX mice may be that infusion of antibiotics induced chronic diarrhea, which eventually led to malnourishment [30]. Overall, our data suggested that ABX treatment not only reduced the total mass of the gut microbes, but also altered their compositions. The antibiotic treatment efficiency was remarkable and led to a significant depletion of the gut microbes.

It is worth noting that SD also impacts the compositions of intestinal microbes in ABX mice. For example, compared with NLD, SD further increased the relative abundance of the Bacteroidetes phylum, but decreased the relative abundance of the Firmicutes phylum, Clostridiales order, Lachnospiraceae family, and Ruminococcaceae family in colonic and cecal contents and epithelium in ABX mice, which indicated that SD still altered the compositions of these low-abundance antibiotic-resistant bacteria, even when the vast majority of gut microbes have been killed by the antibiotics. The probable reason for the increase in the relative abundance of the Bacteroidetes phylum may be that the SD could lead to circadian feeding rhythm disturbances, thus resulting in higher daily energy intake of carbohydrates, total fat, and cholesterol intake, and lower total daily energy expenditure [31]. Another reason may be that SD caused mice to consume more dietary fat, thereby inducing a greater bacterial load that they were less sensitive to and a decrease in antibiotic activity [32]. In addition, others reported that the relative abundance of the colonic Firmicutes phylum and Clostridiales order decreased in rats that were fed a high-fat diet under constant light [33], suggesting that SD promotes overeating and causes negative impacts on the gut microbiota. It is interesting to find that SD also increased the body mass and body temperature in ABX mice compared with NLD, which may be influenced by the alterations in gut microbes. Overall, our data revealed that SD mediated the antagonism between gut microbes and antibiotics, and the greater abundance of the Bacteroides phylum may result in metabolic alterations.

Both the Firmicutes phylum and Bacteroidetes phylum can metabolize carbohydrates in the colon and cecum and produce SCFAs [19]. Results indicated that compared with NLD, SD decreased the concentrations of total SCFAs, and the proportions of butyrate and isobutyrate in the colon contents in ABX mice. However, the alterations of SCFAs in cecal contents were opposite. It has been previously found that the relative abundance of Bacteroidetes was associated with intestinal SCFAs in high-fat-diet fed mice [34]. The increase in the relative abundance of Bacteroidetes in cecal contents may account for the increase in total SCFAs, butyrate, and isobutyrate in the cecum. Meanwhile, the down-regulation of the relative abundance of the Firmicutes phylum may reduce synthesis and metabolism of SCFAs in the colon [35]. As an important microbial metabolite, butyrate is vital to maintain gastrointestinal health due to its ability to enhance epithelial barrier integrity and inhibit inflammation. SD increased the proportion of butyrate in the cecum but decreased in the colon, indicating that the SCFAs’ metabolism was disturbed under SD in ABX mice [36]. Our previous study found that SD did not increase the circulating level of butyrate in the body; therefore, the effects of butyrate on the health of ABX mice need further study. Short day triggered alterations in profiles of intestinal microbes and SCFAs, indicating that the SD may cause systemic metabolic dysfunction. For example, SCFAs affect brain function through interactions with G protein-coupled receptors [37]. Propionate can protect the heart and blood vessels from angiotensin-II-induced damage, butyrate can regulate enteric nervous system (ENS) activity, and acetate-activated *Gpr43* gene also has a significant protective effect on intestinal colorectal cancer in mice [38,39]. Short-chain fatty acids are also considered to synchronize with the peripheral biological clock, and changes in their levels regulate the rhythm of free fatty acid receptor 3 (*Ffar3*) gene expression in the colonic myenteric plexus, controlling normal peristalsis of the colon.

Metabolomics data revealed that SD has a strong impact on colorectal epithelial metabolism. For example, mevalonic acid is an important intermediate product of the mevalonate pathway (MVA), which is closely related to the production of cholesterol [40]. Our data revealed that SD led to a decrease in colorectal epithelial mevalonic acid and serum cholesterol content, indicating SD may impact the synthesis of cholesterol. A set of PLS-DA models has also been previously established based on five metabolites (succinic acid, N2, N2-dimethylguanosine, adenine, citraconic acid, and 1-methylguanosine) with high sensitivity for predicting colorectal cancer [41]. Among them, N2, N2-dimethylguanosine, adenine, and citraconic acid were found to be differentially altered in our work, suggesting that SD may potentially lead to colorectal cancer in mice. Disorders of circadian rhythms can cause disturbances in liver metabolism and corresponding functions and can develop into metabolic diseases in severe cases [42,43]. Thus, our results showed that SD leads to a weakened intestinal epithelial metabolism as a result of the down-regulation of pathways associated with amino acid and fatty acid metabolism in the intestinal epithelium. In turn, these adaptations may contribute to negative effects in the liver. We also noticed that the protective tryptophan metabolism pathway was activated in SD mice, and the possible reason may be that this was a compensatory response of various nutrient metabolism disorders in SD mice. Tryptophan metabolism is driven by intestinal microbes; therefore, another possible reason may be that SD increased relative abundance of some related metabolizing bacteria. However, its impacts on the host’s systemic circulation still need to be further studied. Interestingly, the hepatic transcriptome sequencing data supported this opinion. We found that most DEGs were enriched in hepatic metabolism, endocrine, digestive, and disease processes, indicating that SD may have a series of negative effects in ABX mice. When ABX mice managed under SD, intestinal microbe imbalance caused metabolic disorders, and at the same time induced liver bile acid and pancreatic juice secretion disorders, which further affected digestion. Not only that, but disordered metabolism also affected the synthesis and release of various hormones in the host, such as thyroid hormones, growth hormones, and cortisol. Eventually, various diseases are caused, such as obesity, non-alcoholic fatty liver disease, and even cancer [44,45].

Metabolomics data also revealed that more metabolites were enriched in the purine metabolism pathway in colonic and cecal epithelium under SD in ABX mice; for example, adenine, IMP, adenylsuccinic acid, GDP, GMP, and AMP were decreased while cAMP and cGMP were increased in colonic epithelium. Similarly, inosine, adenine, deoxyguanosine, IMP, GMP, AMP, and hypoxanthine were decreased while cGMP and cAMP were increased in cecal epithelium. In the purine degradation of the uric acid pathway, the synthesizes of intermediate products such as adenine, inosine, and hypoxanthine were decreased, which may lead to a down-regulation in the final product of uric acid. Pathway inhibition may be caused by SD-mediated alterations in gut microbes. As such, it would elicit negative impacts on intestinal metabolism and even lead to inflammation and tumorigenesis. A previous study discussed how the use of antibiotics creates an environment with little competition and allows for overgrowth of fungal populations, up-regulates strains of *Saccharomyces cerevisiae*, and enhances host purine metabolism, leading to an increase in uric acid production [46]. In our work, purine metabolism was decreased in ABX mice under SD, likely due to a decrease in fungi abundance. It also has been reported that intestinal *Lactobacillus* levels were negatively correlated with purine metabolism [47], and enhanced host purine metabolism in germ-free mice may promote the conversion of administered adenine to 2,8-DHA and lead to increased kidney damage [48]. Specific microbial mechanisms need further verification. Moreover, the reduction in these metabolites due to unrestricted proliferation requirements of tumor tissues makes for largely demanding for RNA and DNA, which relates to the changes in the purine metabolism pathway in the tumor environment [49,50]. The abnormal concentrations of purine metabolites are a sensitive indicator of negative energy balance and pathological conditions, including gout, xanthinuria, hyperuricemia, and renal failure [51], and xanthine is also a potential tumor marker [52]. SD may reduce purine metabolism in the absence of the barrier function of the gut microbes and their metabolites, and further may lead to potential tumors of the host.

There are two major limitations to our study. First, the detection of intestinal microbes is based on the relative abundance, and absolute abundance may provide more references. Second, the gut metabolomics as well as hepatic transcriptome data lack comparisons in NC mice under NLD and SD, which will be refined in subsequent experiments.

## 4. Materials and Methods

### 4.1. Ethics

All animal experiments were performed according to the ethical policies and procedures approved by the Animal Care and Use Committee of Yangzhou University, Jiangsu, China (approval no. SYXK (Su) 2017-0044).

### 4.2. Mice Management and Experimental Design

The C57BL/6N murine line was selected and purchased from GemPharmatech Co., Ltd. (Nanjing, China). This is a widely-used model for intestinal microbe and metabolism research [53]. All mice were initially placed in the same cage to harmonize the diet and gut microbes. Mice were then randomly divided into a standard feeding (NC) group and treated with compound antibiotics (ABX) with alternating photoperiods of normal light–dark cycle (NLD, 12 h light/12 h dark) and short day (SD, 8 h light/16 h dark), referring to previous reports [54,55], with each group containing 6 mice. The initial body weight of each C57BL/6 mouse was 19–20 g, with no differences among the groups. Mice in the antibiotic-treated groups were first placed into Lugol’s iodine solution (2 g potassium iodide and 1 g iodine tablets dissolved into 30 L sterilize water) at 39 °C for 3 s to disinfect the skin [56]. They were then fed sterilized water with compound antibiotics containing 100 μg/mL gentamicin (for Gram-negative intestinal bacteria), 2.5 mg/mL sulbenicillin and cephalosporin (for Gram-positive intestinal bacteria), and 30 μg/mL amphotericin B (for intestinal fungi) in an aseptic environment. Antibiotics were purchased from Qingdao Jisskang Biotechnology Co., Ltd. This cocktail of broad-spectrum antibiotics can effectively disrupt intestinal microbiota homeostasis [25,26,27]. Each mouse was managed in a single cage in an environmentally controlled warehouse that allowed for manipulating light–dark cycles according to regulations. The light–dark cycles were controlled with an LED light strip of 150 to 200 lx and a temperature of 4500 to 5000 K. Temperature within the warehouse was 22–24 °C, relative humidity was 55–65%, and negative pressure ventilation was used. Mice were kept under strict light–dark cycles, with lights being turned on at 6 a.m. and turned off at 6 p.m. of NLD cycle treatment, while lights were turned on at 6 a.m. and turned off at 2 p.m. of SD treatment. The trough, drinking fountain, and litter were changed every day after the lights were turned on at 6 a.m. Adequate sterilized drinking water and pelleted feed were provided (sterilized by Co_60_ irradiation). The composition of the commercial maintenance diet consisted of corn, soybean, wheat, chicken meal, fish meal, vegetable oil, and a variety of customized vitamins and trace elements. The experimental period was 42 days and was divided into 2 stages. The first stage lasted 28 days and was used for diet, LD cycles, and antibiotic acclimatization, and the next stage lasted 14 days and was used for formal trial under strict environmental control conditions.

### 4.3. Sample Collection

All mice were fasted for 24 h before sampling and the body weight was recorded. Blood was collected from the retroorbital sinus for measurement of serum biochemical indices. Mice were then anesthetized with ether and euthanized by spinal dislocation from 10:00–12:00 a.m. The colon and cecum tissues were harvested, rinsed in phosphate buffer solution (PBS), and rapidly frozen in liquid nitrogen. Colonic and cecal contents were stripped along the outer wall of the intestine and stored in liquid nitrogen. The liver, thymus, and spleen were also isolated, weighed after blotting excess blood, and samples were immediately snap-frozen in liquid nitrogen until analysis. The organ index was calculated using the Equation (1) [57]:

Organ index = organ mass (g)/mouse body mass (g) × 100(1)

### 4.4. Detection of Serum Biochemical Parameters

Blood samples were centrifuged at 4500× *g* and 4 °C for 10 min to collect the serum. Then, the serum concentrations of alanine aminotransferase (ALT) (BC1555, Beijing Solarbio Science & Technology Co., Ltd., Beijing, China), aspartate aminotransferase (AST) (BC1565, Solarbio), glucose (GLU) (BC2505, Solarbio), total cholesterol (TC) (BC1985, Solarbio), high-density lipoprotein (HDL-C) (A112-1-1, Nanjing Jiancheng Bioengineering Institute, Nanjing, China), and low-density lipoprotein (LDL-C) (A113-1-1, Jiancheng) were detected following the manufacturer’s protocols.

### 4.5. Determination of SCFAs

Concentrations of acetate, propionate, isobutyrate, butyrate, isovalerate, and valerate in colonic and cecal contents were determined following previous protocols [58]. Briefly, 0.5 g of colonic and cecal contents from each group were used. Ultrapure water at 1.25 mL per sample was mixed with contents, vortexed for 5 min, and then centrifuged at 13,400× *g* and 4 °C for 10 min to collect the supernatant and filter through a 0.25 μM syringe filter. A total of 0.4 mL metaphosphoric acid (contained 20% of 60 mM crotonic acid as internal standard) was added, and the entire contents were vortexed, centrifuged, and filtered, and the supernatant was collected for subsequent analyses. A standard mix (0.4 μL) containing 55.06 mM acetate, 15.84 mM propionate, 4.25 mM isobutyrate, 8.57 mM butyrate, 3.58 mM isovalerate, and 3.56 mM valerate and test samples were mixed and run through a CP-WAX capillary column (length 30 m, inner diameter 0.53 mm, and film thickness 1 μm) in a gas chromatograph (GC-9A, Shimadzu, Kyoto, Japan). Program settings and calculations of concentrations were according to a previous protocol [59].

### 4.6. Colonic and Cecal Contents and Epithelial DNA Extraction

A 0.5 g sample of colonic and cecal contents from each group was used for total gDNA extraction with the TIANamp Stool DNA Kit (DP328, Tiangen, Beijing, China) according to the manufacturer’s instructions. In addition, 50 mg of colonic and cecal epithelium samples was grounded for total gDNA extraction with the FastPure Cell/Tissue DNA Isolation Mini Kit (DC102-01, Vazyme, Nanjing, China) following the manufacturer’s protocols. The concentration and purity of total gDNA were determined with a NanoDrop spectrophotometer (Thermo Fisher Scientific, Waltham, MA, USA). All gDNA samples were diluted to a unified concentration of 80 ng/μL and stored at −80 °C for the determination of the relative abundance of gut microbes by RT-PCR.

### 4.7. Quantitative Real-Time PCR of Gut Microbes

Extracted gDNA samples were used as templates for quantitative real-time PCR (RT-qPCR) using the 2× TSINGKE Master qPCR Mix (SYBR Green I) (TSE201, Tsingke, Beijing, China) in an ABI7500 (Thermo Fisher Scientific, Waltham, MA, USA) sequence detector. Three replicates per sample in each pair of primers were examined. PCR mixtures included 10 μL 2 × Mix, 0.4 μL forward primer (10 μM), 0.4 μL reverse primer (10 μM), 1 μL template gDNA (80 ng/μL), and 8.2 μL distilled H_2_O. The PCR cycling conditions were as follows: 95 °C for 30 s, 40 cycles of 95 °C for 10 s, and 60 °C for 30 s, ultimately tested at 95 °C for 15 s, 60 °C for 60 s, and 95 °C for 15 s. The standard curve method and QuantStudio™ 7 Flex Real-Time PCR Software (Applied Biosystems, Foster, CA, USA) were used for data analysis. Relative abundance of gut microbes was determined using the 2^−ΔΔCt^ method [60], where the 16S rRNA gene amplified by the total bacterial primer set was used as an internal reference [61,62]. Thus, the standard (NLD) group was considered as a control, and Fold Changes in abundance were expressed relative to the standard (NLD) group. Primer sets for quantification of the Bacteroidetes phylum, Firmicutes phylum, Clostridiales order, Lachnospiraceae family, and Ruminococcaceae family were designed and synthesized by Sangon Biotech Co., Ltd., Shanghai, China, (Table 1).

### 4.8. Metabolite Extraction in Intestinal Contents and Epithelium

One hundred milligrams colonic and cecal contents and epithelium from each group was ground with liquid nitrogen for metabolite extraction. The homogenate was resuspended with a 500 μL extraction solution (80% methanol and 0.1% formic acid) and vortexed. Samples were then centrifuged at 16,750× *g* and 4 °C for 20 min. Subsequently, samples were incubated for 5 min on ice and 200 μL of supernatant diluted to a final concentration containing 53% methanol using LC-MS grade water. Samples were subsequently transferred to a fresh Eppendorf tube and then centrifuged at 15,000× *g* at 4 °C for 20 min. Lastly, the supernatant was injected into the LC-MS/MS system for further analysis, with a 10 μL aliquot of supernatant from each sample mixed as QC samples [65].

### 4.9. Non-Targeted Metabolomics Analysis

LC/MS analyses were performed using a Vanquish UHPLC system (Thermo Fisher Scientific) coupled with an Orbitrap Q Exactive^TM^ HF mass spectrometer (Thermo Fisher Scientific) in Novogene Co., Ltd. (Beijing, China). A 1 μL of sample analyte was injected into a Hypesil Goldcolumn (100 × 2.1 mm, 1.9 μm) using a 17 min linear gradient at a flow rate of 0.2 mL/min. The eluents for the positive polarity mode were eluent A (0.1% formic acid in water) and eluent B (methanol), and the eluents for the negative polarity mode were eluent A (5 mM ammonium acetate at pH 9.0) and eluent B (methanol). The solvent gradient run program was as follows: 2% B, 1.5 min; 2–100% B, 12.0 min; 100% B, 14.0 min; 100–2% B, 14.1 min; 2% B, 17 min. The QExactive^TM^ HF mass spectrometer was operated in both positive and negative polarity mode with a spray voltage of 3.2 kV, capillary temperature of 320 °C, sheath gas flow rate of 40 arb, and aux gas flow rate of 10 arb.

The raw data files generated by LC/MS were further processed with the Compound Discoverer 3.1 (CD3.1, Thermo Fisher Scientific) software, including peak detection, peak alignment, peak grouping, retention time normalization, and peak integration. The main parameters were set as follows: retention time tolerance, 0.2 min; actual mass tolerance, 5 ppm; signal intensity tolerance, 30%; ratio of signal to noise ratio, 3; and minimum intensity 100,000. Then, the molecular formula was predicted via additive ions, molecular ion peaks, and fragment ions after total spectral intensity was normalized. Accurate qualitative and relative quantitative profiles of metabolites were obtained after matching the peaks with mzCloud (https://www.mzcloud.org/, (accessed on 25 February 2021)), mzVault, and MassList database according to a previously reported protocol by our laboratory [66]. Data analyses were performed using the statistical software R (R version R-3.4.3), Python (Python 2.7.6 version), and CentOS (CentOS release 6.6). When data were not normally distributed, normal transformations were attempted using of area normalization method. Metabolites were annotated via the Kyoto Encyclopedia of Genes and Genomes (KEGG) database (https://www.genome.jp/kegg/pathway.html, (accessed on 25 February 2021)) and the Human Metabolome Database (HMDB) (https://hmdb.ca/metabolites, (accessed on 25 February 2021)).

For metabolomics data, principal components analysis (PCA) and partial least squares discriminant analysis (PLS-DA) were performed using metaX (a flexible and comprehensive software for processing metabolomics data). The Hotelling’s T2 region demonstrated an ellipse in the model score, which was defined at a 95% confidence interval for model variation. Analysis of variance (ANOVA) was conducted using SPSS software (version 16.0) to calculate statistical significance (*p*-value). The variable importance in projection (VIP) was calculated based on the PLS-DA model to identify significant metabolites. Differential metabolites were considered as VIP > 1 and *p* < 0.05. Volcano plots were used to filter metabolites of interest based on log2 (Fold Change) and −log10 (*p*-value). The cluster maps of differentially altered metabolites were drawn using R software and the “Pheatmap” package. The biological functions and metabolic pathways were enriched in the KEGG database with MetaboAnalyst 5.0 website (https://www.metaboanalyst.ca/MetaboAnalyst/ModuleView.xhtml, accessed on 20 July 2021).

### 4.10. Hepatic Transcriptome Sequencing and Data Processing

RNA-Seq was performed as described previously before [67]. Briefly, total RNA for hepatic transcriptome sequencing was isolated and purified using TRIzol reagent (Invitrogen, Carlsbad, CA, USA) following the manufacturer’s procedure. The RNA amount and purity were quantified using NanoDrop ND-1000 spectrophotometer. The RNA integrity was assessed by Agilent 2100 with RIN number > 7.0. The cDNA library was constructed and then sequenced on Illumina Novaseq 6000 sequencing platform (Illumina, San Diego, CA, USA) by Novogene Co., Ltd. (Beijing, China).

The Fastp software (v0.23.1) [68] was used to perform quality control on the raw data and obtain clean data. The HISAT2 software (v2.1.0) [69] was used to align the obtained clean data to the reference genome (Mus musculus genome, GRCm39). The Samtools software (v.1.10) was used to sort and convert the SAM files to BAM format [70]. The Stringtie (v2.2.1) software was used to assemble and quantify the transcripts and genes based on read counts [69]. Finally, the expression levels of all the mRNA and the identification of differentially expressed genes (DEGs) were estimated using DESeq2 package (1.36.0) in R (v4.2) software. Genes that passed a threshold of *p* < 0.05 and |log_2_ Fold Change| > 1 in DEG analysis were considered for further analysis. Functional enrichment was analyzed and visualized using KEGG database. The hierarchical clustering was generated using Pheatmap package (v.1.0.12).

### 4.11. Statistical Analysis

All data are expressed as mean ± standard error means (SEM). A Student’s *t*-test was carried out, and *p* < 0.05 was considered statistically significant. GraphPad Prism 6.0 software was used to draw the histograms. Correlations between selected parameters and metabolites were calculated using Pearson’s correlation coefficients and visualized with GraphPad Prism and R software “Pheatmap” package.

## 5. Conclusions

Antibiotics can efficiently alter intestinal microbe and metabolite profiles, thus negatively impacting the homeostasis of the intestine. Compared with NLD, SD altered intestinal microbes and their metabolism in ABX mice. Moreover, SD increased the abundance of the Bacteroidetes phylum, while it decreased the relative abundances of the Firmicutes phylum, Clostridiales order, Lachnospiraceae family, and Ruminococcaceae family in intestines, and further induced intestinal SCFA metabolism disorders. Not only that, SD also induced epithelial and hepatic purine metabolism pathway imbalance in ABX mice, the gut microbes, and their metabolites, all of which could contribute to host metabolism and digestion, endocrine system disorders, and may even cause diseases in the host.

## Figures and Tables

**Table 1:** The primer sequences of mice gut microbes.

Gut Microbes	Forward Primer Sequence	Reverse Primer Sequence	References
Firmicutes phylum	GGAGYATGTGGTTTAATTCGAAGCA	AGCTGACGACAACCATGCAC	[63]
Bacteroidetes phylum	GGARCATGTGGTTTAATTCGATGAT	AGCTGACGACAACCATGCAG	[63]
Clostridiales order	GCGTTATCCGGATTTAC	ACACCTAGTATTCATCG	[64]
Lachnospiraceae family	CGGTACCTGACTAAGAAGC	AGTTTTATTCTTGCGAACG	[64]
Ruminococcaceae family	TTAACACAATAAGTWATCCACCTGG	ACCTTCCTCCGTTTTGTCAAC	[64]
Total bacteria	CGGCAACGAGCGCAACCC	CCATTGTAGCACGTGTGTAGCC	[62]

## Data Availability

The transcriptome sequencing data have been deposited in NCBI Gene Expression Omnibus database under accession code GSE203566. Additional data related to this paper may be requested from the authors.

## Supplementary Materials

The following supporting information can be downloaded at: https://www.mdpi.com/article/10.3390/ijms23116008/s1.
