# Supplementary material for: The Short-Day Cycle Induces Intestinal Epithelial Purine Metabolism Imbalance and Hepatic Disfunctions in Antibiotic-Mediated Gut Microbiota Perturbation Mice
Source: Int J Mol Sci. 2022 May 26;23(11):6008. doi: 10.3390/ijms23116008 (PMC9181120; doi:10.3390/ijms23116008)
Supplement: Supplementary file 1 [file ijms-23-06008-s001.zip › ijms-1710152-supplementary.pdf]

Table S1. The significantly different metabolites in Metabolomics of SD *vs.* NLD in colonic content and epithelium of antibiotic-treated mice

Classification	Metabolites	FC	*P*	VIP	Trend
	**Colonic content**				
Amino acid	L-Phenylalanine	2.21	0.03	4.03	up
Carbohydrate	N-Acetylneuraminic acid	2.13	0.02	4.22	up
Fatty acid	Mevalonic acid	0.51	0.01	2.22	down
Purine	Phenylglyoxylic acid	1.81	0.03	1.45	up
	**Colonic epithelium**				
Amino acid	2-Aminoadipic acid	0.56	0.04	1.09	down
Amino acid	N-Acetylglycine	0.61	0.05	1.47	down
Amino acid	O-Phospho-L-serine	0.51	0.04	1.24	down
Amino acid	Pyroglutamic acid	0.48	0.01	1.46	down
Carbohydrate	1,5-Anhydro-D-glucitol	0.66	0.01	1.33	down
Carbohydrate	D-Glucose 6-phosphate	2.79	0.04	1.05	up
Carbohydrate	D-Threose	0.53	0.04	1.44	down
Carbohydrate	N-Acetylgalactosamine 4-sulphate	0.43	0.00	1.52	down
Fatty acid	2-Methylpentanedioic acid	0.53	0.03	1.59	down
Fatty acid	3-Hexenedioic acid	0.49	0.01	1.88	down
Fatty acid	3-Methyladipic acid	0.55	0.03	1.73	down
Fatty acid	Citraconic acid	0.32	0.01	1.25	down
Fatty acid	Erucic acid	0.31	0.02	2.19	down
Fatty acid	Mevalonic acid	0.49	0.05	1.31	down
Fatty acid	Octanedioic acid	0.59	0.04	1.09	down
Fatty acid	Tetradecanedioic acid	0.60	0.02	1.56	down
Fatty acid	Undecanedioic acid	0.48	0.02	1.40	down
Purine	cAMP	3.39	0.01	2.51	up
Purine	cGMP	2.50	0.02	2.40	up
Purine	N2, N2-Dimethylguanosine	3.43	0.05	3.16	up
Purine	AMP	0.05	0.01	1.19	down
Purine	GDP	0.30	0.01	1.71	down
Purine	GMP	0.21	0.01	1.39	down
Purine	IMP	0.15	0.01	3.41	down

Purine	Hydroxyadenine	3.01	0.01	2.55	up
Purine	Adenine	0.24	0.01	4.38	down
Pyrimidine	Orotidine	0.09	0.01	1.98	down
Pyrimidine	UDP	0.13	0.01	1.07	down
Pyrimidine	UDP Glucuronic Acid	0.06	0.01	1.86	down
Pyrimidine	UMP	0.19	0.01	1.89	down
Others	Adenylsuccinic acid	0.05	0.02	2.33	down
Others	dCDP	0.28	0.01	1.67	down

FC, fold change of differential metabolites of SD vs. NLD; VIP, variable importance in the projection value from PLS-DA model. Up and down indicated that the metabolites were up-regulated and down-regulated under SD vs. NLD in colonic content and epithelium.

Table S2. The significantly different metabolites in Metabolomics of SD *vs.* NLD in cecal content and epithelium of antibiotic-treated mice

Classification	Metabolites	FC	*P*	VIP	Trend
	**Cecal content**				
Amino acid	Cinnamoylglycine	0.55	0.03	3.22	down
Amino acid	gamma-Glutamylleucine	0.50	0.02	1.19	down
Amino acid	Glutathione	1.87	0.01	2.46	up
Amino acid	L-cysteine	0.27	0.02	2.58	down
Amino acid	L-Histidine	2.67	0.03	4.22	up
Amino acid	N-Lactoylphenylalanine	2.14	0.04	2.26	up
Amino acid	Phenylalanylphenylalanine	0.41	0.04	2.51	down
Amino acid	Tyrosyl-Tyrosine	0.38	0.04	3.17	down
Carbohydrate	N-Acetylneuraminic acid	1.51	0.03	2.28	up
Fatty acid	Nervonic acid	4.56	0.04	3.15	up
Purine	Adenine	0.67	0.01	1.56	down
	**Cecal epithelium**				
Amino acid	Glutathione	0.51	0.01	1.57	down
Amino acid	Hydroxyproline	0.49	0.03	1.23	down
Amino acid	Lanthionine	0.44	0.01	1.11	down
Amino acid	L-cysteine	0.04	0.01	2.13	down
Amino acid	L-Histidine	1.53	0.01	1.31	up
Amino acid	Methionine sulfoxide	0.64	0.02	1.56	down
Amino acid	N-Acetylmethionine	0.40	0.01	1.16	down
Amino acid	Pantetheine	0.46	0.01	1.08	down
Amino acid	Tyrosyl-Tyrosine	0.43	0.02	1.68	down
Carbohydrate	6'-Sialyllactose	2.98	0.02	1.70	up
Carbohydrate	D-Maltose	0.22	0.05	1.32	down
Carbohydrate	Glyceraldehyde 3-phosphate	0.47	0.04	1.03	down
Carbohydrate	Threonic acid	0.34	0.02	1.30	down
Fatty acid	20-Carboxy-Leukotriene B4	5.31	0.02	2.02	up
Fatty acid	3-Hexenedioic acid	0.55	0.02	1.30	down
Fatty acid	3-Methyladipic acid	0.58	0.03	1.26	down
Fatty acid	Docosatrienoic acid	24.14	0.03	1.60	up
Fatty acid	Elaidic acid	2.96	0.01	1.14	up

Fatty acid	Ethyl dodecanoate	3.03	0.03	1.31	up
Fatty acid	Mevalonic acid	0.48	0.03	1.18	down
Fatty acid	Stearic acid	4.29	0.01	1.30	up
Fatty acid	Undecanedioic acid	0.49	0.02	1.22	down
Fatty acid	Azelaic acid	0.57	0.04	1.17	down
Purine	2-Hydroxyadenine	2.34	0.03	1.56	up
Purine	Adenine	0.39	0.01	2.24	down
Purine	cAMP	2.07	0.01	1.19	up
Purine	cGMP	8.97	0.01	3.54	up
Purine	Deoxyguanosine	0.37	0.03	1.66	down
Purine	Inosine	0.54	0.03	2.46	down
Purine	IMP	0.31	0.01	1.27	down
Purine	Succinyladenosine	0.27	0.01	1.48	down
Pyrimidine	5-Methyldeoxycytidine	0.47	0.02	1.21	down
Pyrimidine	Citicoline	0.38	0.05	1.06	down
Pyrimidine	UDP	0.18	0.01	1.04	down

FC, fold change of differential metabolites of SD vs. NLD; VIP, variable importance in the projection value from PLS-DA model. Up and down indicated that the metabolites were up-regulated and down-regulated under SD vs. NLD in cecal content and epithelium.
